# Supplementary material for: Long-Term Use of Amoxicillin Is Associated with Changes in Gene Expression and DNA Methylation in Patients with Low Back Pain and Modic Changes
Source: Antibiotics (Basel). 2023 Jul 21;12(7):1217. doi: 10.3390/antibiotics12071217 (PMC10376514; doi:10.3390/antibiotics12071217)
Supplement: Supplementary file 1 [file antibiotics-12-01217-s001.zip › Supplementary_figures.pdf]

Supplementary figures

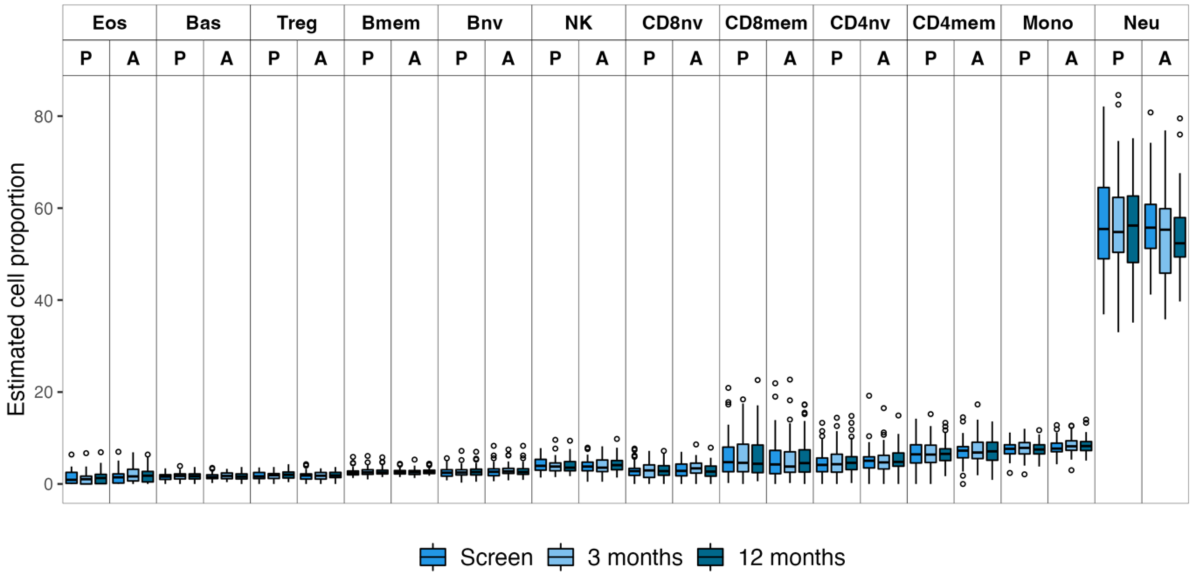

Supplementary Figure S1: Box plots of estimated cell type proportions (n=12 cell types) in patients in placebo (P) and antibiotics (A) groups from all time points based on DNA methylation data. Eos: Eosinophils; Bas: Basophils; Treg: Regulatory T cells; Bmem: Memory B cells; Bnv: Naïve B cells; NK: Natural killer cells; CD8nv: Naïve cytotoxic T cells; CD8mem: Memory cytotoxic T cells; CD4nv: Naïve helper T cells; CD4mem: Memory helper T cells; Mono: Monocytes; Neu: Neutrophils

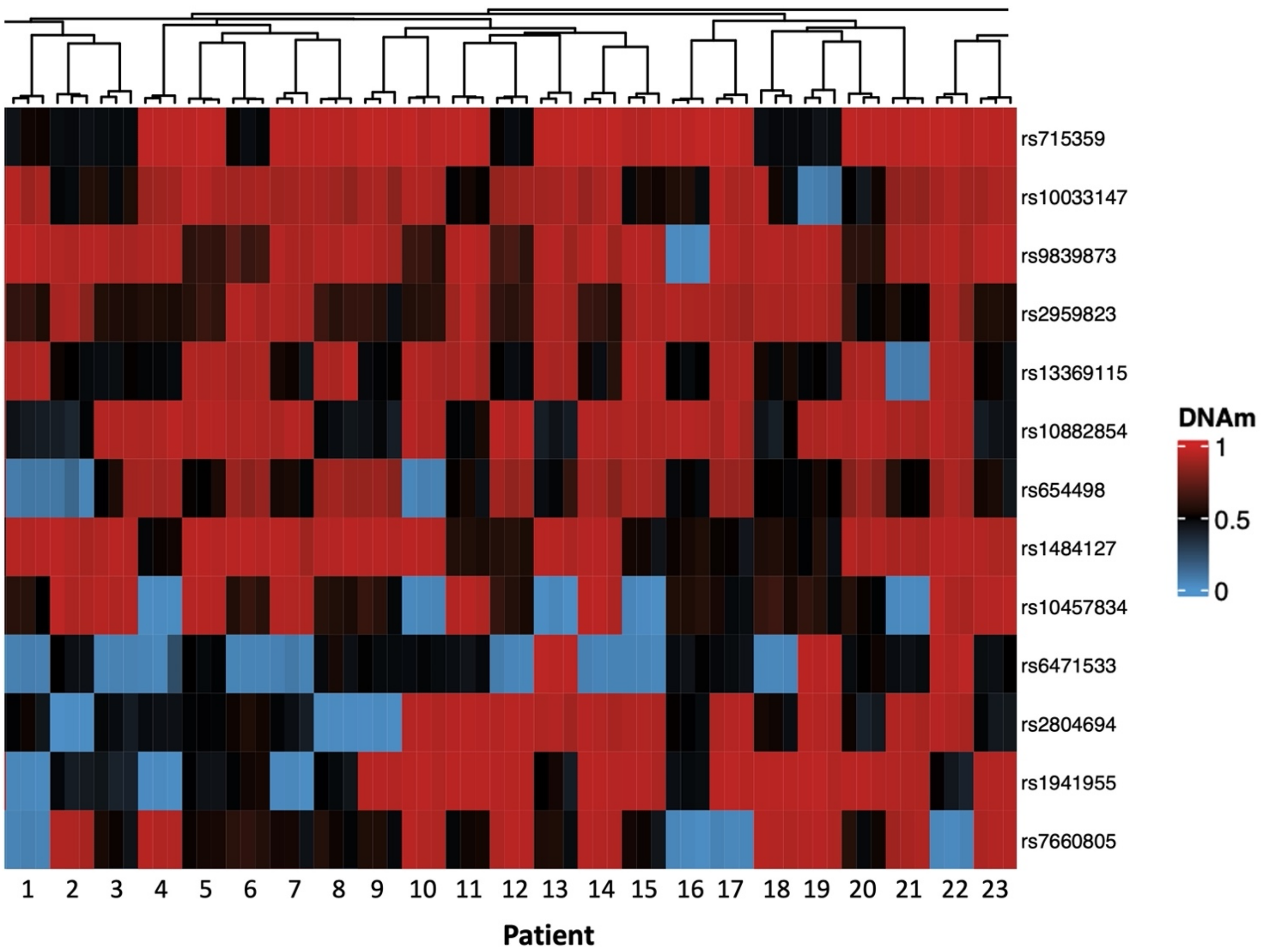

Supplementary Figure S2: Heatmap of DNA methylation at control probes (n=59) from each patient at each time point demonstrates correct intra-individual clustering of time points. Each group of three consecutive columns belongs to the same individual. Only a selection of samples/probes are shown.

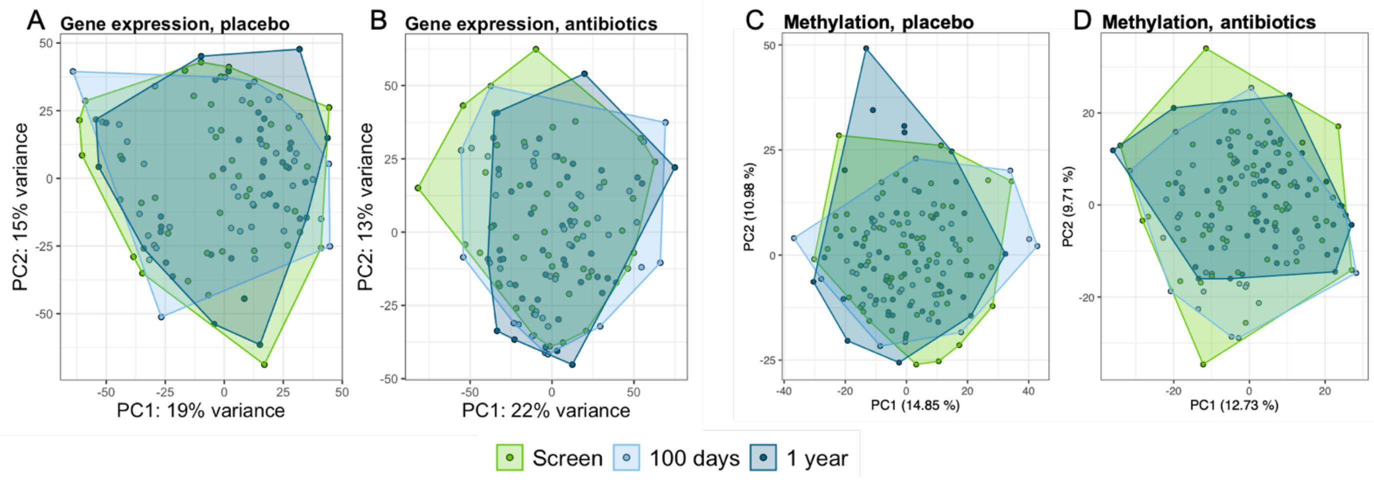

Supplementary Figure S3: Principal component plots comparing the three time points.

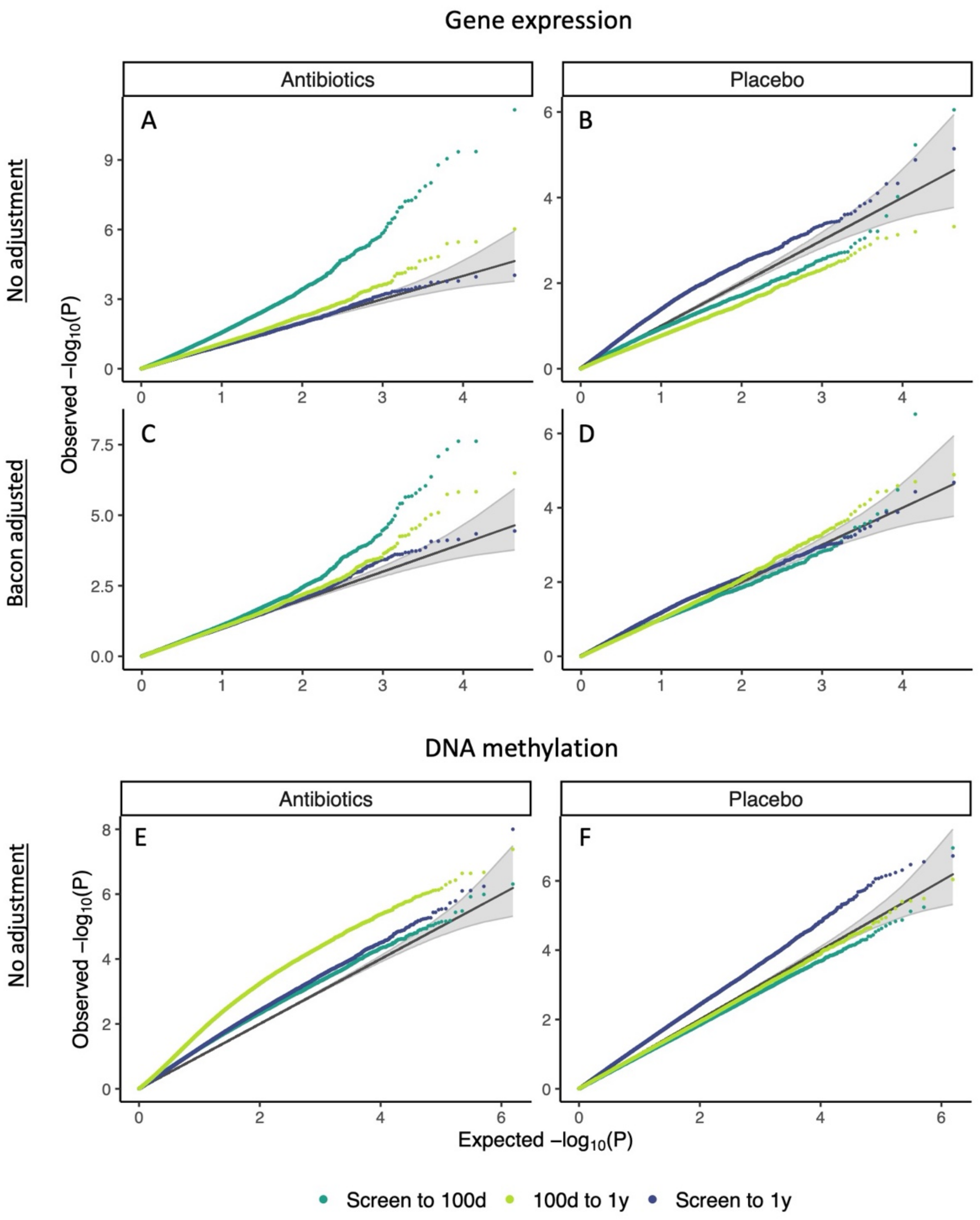

Supplementary Figure S4: Q-Q plots from all analyses in the two treatment groups: A-B) Gene expression analysis, no adjustment of p-values. C-D) Gene expression analysis, after bacon adjustment of p-values. E-F) Methylation analysis, no adjustment of p-values.

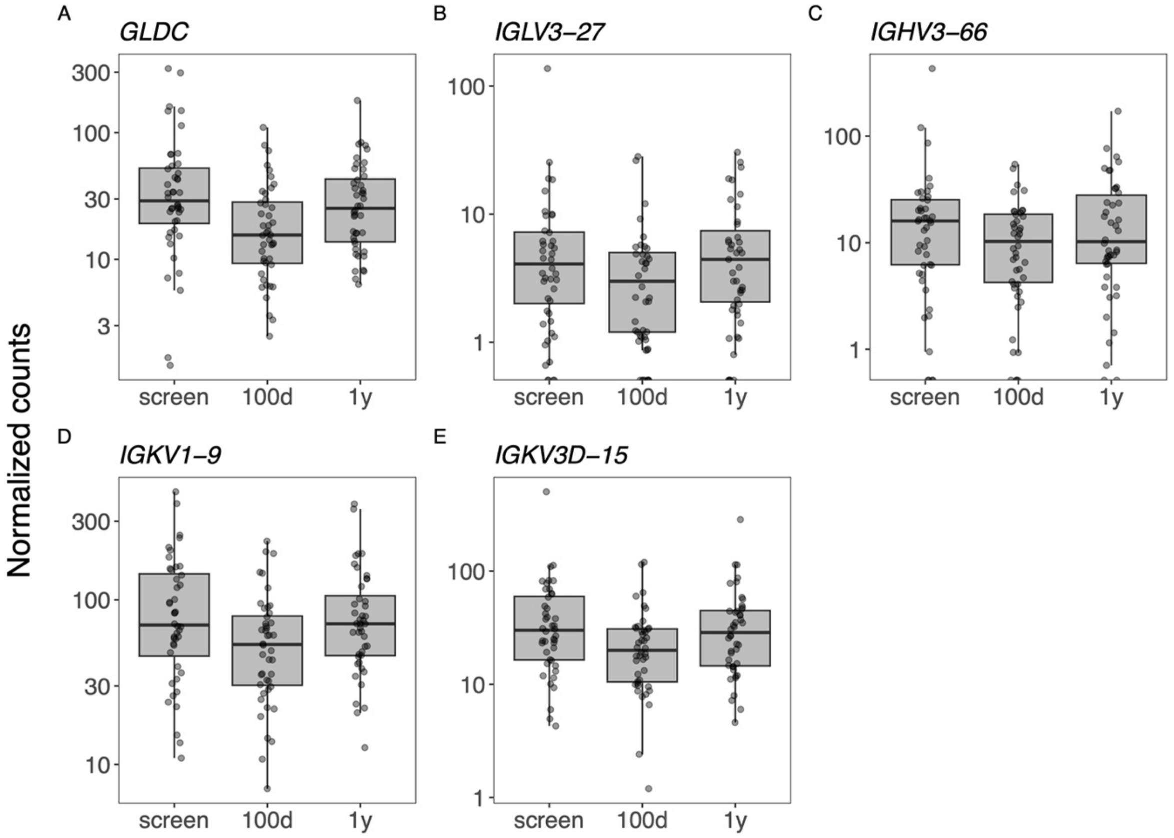

Supplementary Figure S5: Expression of genes that were significantly differentially expressed during both time intervals in the antibiotics group.
